# *Xiangbin* prescription for the recovery of gastrointestinal function after abdominal surgery (the XBPRS trial): study protocol for a randomized controlled trial

**DOI:** 10.1186/s13063-018-2484-z

**Published:** 2018-02-27

**Authors:** Huachan Gan, Jinxuan Lin, Zhi Jiang, Qicheng Chen, Lixing Cao, Zhiqiang Chen

**Affiliations:** The Second Affiliated Hospital of Guangzhou University of Chinese Medicine, Guangzhou, 510120 China

**Keywords:** *Xiangbin* prescription, Perioperative period, Gastrointestinal function, Fast recovery, Randomized parallel controlled trial

## Abstract

**Background:**

Most patients who undergo abdominal surgery recover bowel movements within a week; however, some suffer prolonged intestinal paralysis or postoperative ileus (POI) leading to complications, such as infection and intestinal adhesions, which can extend hospitalization and increase readmission rates, and consequently increasing healthcare costs. Chinese medicine is effective for accelerating the recovery of gastrointestinal function after abdominal surgery. *Xiangbin* prescription (XBP) is the standard prescription for this purpose in our hospital; however, randomized controlled trials of it have not yet been conducted.

**Methods/design:**

This double-blind, randomized controlled clinical trial aims to recruit patients who have undergone abdominal surgery and experienced postoperative dysmotility to evaluate the efficacy and safety of XBP for preventing POI and accelerating recovery. The research will tackle the common problem of slow recovery of gastrointestinal function after surgery. The participants will be patients who undergo laparoscopic radical resection of rectal carcinoma or laparoscopic panhysterectomy of a benign lesion. Primary outcome measures will be time to first flatus, defecation, normal bowel sounds, and liquid/semi-liquid/general diet. Good Clinical Practice (GCP) standards of efficacy and safety will also be evaluated, along with objective investigation of the mechanism of action of ghrelin.

**Discussion:**

This pivotal trial will be a standardized, scientific, clinical trial designed to evaluate the use of XBP for the recovery of gastrointestinal function after surgery, and it will conform to international standards for clinical trials for the recognition of traditional Chinese medicine.

**Trial registration:**

Chinese Clinical Trial Registry, ID: ChiCTR-TRC-14004156. Registered on 3 January 2014.

**Electronic supplementary material:**

The online version of this article (10.1186/s13063-018-2484-z) contains supplementary material, which is available to authorized users.

## Background

It has been shown that the small intestine recovers more quickly after surgery (0–24 h) than the stomach (24–48 h) and colon (48–72 h) [1]. Postoperative ileus (POI) can occur as a common complication of anesthesia and surgery after various types of abdominal surgery [2]. The neuronal reflex required for gastrointestinal motility can be temporarily inhibited during surgery, causing nausea, vomiting, abdominal distension, and delaying the postoperative time to first flatus and defecation [3]. Numerous clinical research studies have shown that failure to restore adequate bowel function can result in a series of complications, including infection and intestinal adhesion, leading to prolonged hospital stay, and increased costs and readmission rates. The incidence of POI after abdominal surgery is 10–30% [4]. Reliable data demonstrate that the total cost of surgery can double for patients with, compared with those without, POI ($16,612 vs. $8316; *p* < 0.05) [5]. The Chinese herbal medicine *Xiangbin* prescription (XBP) has been proven to accelerate the recovery of bowel function [6]; however, its use lacks compliance with Good Clinical Practice standards. To extend and ensure the appropriate use of traditional Chinese medicine (TCM), rigorous studies are needed to ensure the safe and effective use of Chinese herbal medicines for the improvement of gastrointestinal motility.

XBP is prescribed as standard in our hospital to promote recovery of gastrointestinal function after abdominal surgery. The function of XBP has been determined based on previous clinical practice, which has demonstrated that the prescription can safely and effectively restore gastrointestinal motility after abdominal surgery. We have conducted numerous research studies into gastrointestinal motility in recent years, and we consider that anesthesia and surgery lead to the POI, referred to as the syndromes, *Qi* deficiency and *Qi* stagnation, in Chinese medicine. XBP is a compound of *Panax ginseng* (*Renshen*), *Areca catechu* L. (*Binlang*), *Prunus persica* Batsch (*Taoren*), *Lindera aggregata* Kosterm (*Wuyao*), and *Fructus amomi* (*Sharen*). *P. ginseng* (*Renshen*) is an edible Chinese herb that promotes the production of body fluids and has a tranquilizing effect, and ginseng oligopeptides (GOP), small-molecular oligopeptides isolated from ginseng, have been proven to have anti-fatigue effects [7]. *A. catechu* L. (*Binlang*) has the function in Chinese medicine of promoting the circulation of *Qi*, and there is evidence that, one of its components, arecoline, affects gastrointestinal motility by direct binding to M receptors [8]. *P. persica* Batsch (*Taoren*) has the function in Chinese medicine of improving blood circulation and relaxing the bowels and there is evidence that its extract can lower blood viscosity [9]. *L. aggregata* Kosterm (*Wuyao*) has the function in Chinese medicine of promoting *Qi* circulation to relieve pain and one of its components. Total alkaloids have anti-inflammatory and analgesic effects [10]. *F. amomi* (*Sharen*) has the functions in Chinese medicine of removing dampness to restore the normal function of the stomach, while its water extract has a role in promoting gastrointestinal peristalsis [11].

Here, we describe a protocol for a double-blind, randomized controlled, clinical trial to evaluate the safety and efficacy of the use of XBP to promote gastrointestinal function recovery after abdominal surgery.

## Methods/design

This pivotal study has been designed as a double-blind, randomized controlled, clinical trial. Patients who have undergone laparoscopic radical resection of rectal carcinoma or laparoscopic panhysterectomy for benign lesions, between March 2015 and June 2018 at Guangdong Provincial Hospital of Traditional Chinese Medicine, will be selected and invited to participate. Patients will be randomly assigned to either the XBP group or the placebo group using numbers randomly generated by the webtool http://www.gztcmgcp.com/sjxt/login.asp. The efficacy and safety of XBP for patients who have undergone abdominal surgery will be evaluated by comparing various indices in the two different groups, including: time to first passage of flatus, first defecation, normal bowel sounds, and consumption of a liquid/semi-liquid/general diet. The study aim is to determine whether XBP can improve the speed of restoration of postoperative gastrointestinal motility. The study protocol has been approved by the Ethical Committee of the Second Affiliated Hospital of Guangzhou University of Traditional Chinese Medicine (Guangdong Provincial Hospital of Traditional Chinese Medicine). Written informed consent was obtained from all volunteers, and the study conformed to the ethical principles set forth by the Declaration of Helsinki. The study protocol is registered at the Chinese Clinical Trial Registry (http://www.chictr.org.cn/showproj.aspx?proj=5412).

### Participants

Participants who have undergone laparoscopic radical resection of rectal carcinoma in the Gastrointestinal Surgery Department or laparoscopic panhysterectomy of benign lesions in the Gynecology Department of Guangdong Provincial Hospital of Traditional Chinese Medicine will been recruitment. The inclusion, exclusion, and elimination criteria are listed in Table 1.

**Table 1:** Inclusion, exclusion, and elimination criteria

Category	Criteria
Inclusion criteria	Patients who have undergone surgery for gastric or colorectal cancer, or hysterectomy for hysteromyoma Age 40–75 years old Duration of surgery, 1–4 h Time under anesthesia, 1.5–4.5 h TCM syndrome, Qi deficiency and Qi stagnation Provision of signed, informed consent
Exclusion criteria	Advanced malignant tumor cachexia, extreme weakness Malignant tumors requiring extended radical surgery Cardiovascular, liver, kidney, brain, or lung co-morbidity, or other serious diseases Poorly controlled hypertension or diabetes Mental disability Allergy to the intervention Pregnant or breast-feeding women Severe malnutrition (serum albumin < 21 g/L, pre-albumin < 0.1 g/L) Repeated abdominal surgery and severe intestinal adhesions Blood loss > 400 mL during surgery Serious complications occurring within 6 h after surgery (e.g., multiple organ dysfunction) Postoperative intraperitoneal hyperthermic perfusion Emergency surgery Prokinetic drugs prescribed after surgery Patient participating in another clinical study, or having participated in another clinical study in the previous month Patient considered inappropriate to participate the study by clinical investigators
Elimination criteria	Do not meet the inclusion criteria, but mistakenly included Worsening condition that cannot be controlled in 3 days Other complications because of interventions, including severe allergy or serious adverse events Serious complications, infection, or requiring a second operation during treatment Refusal to continue the treatment, regardless of the reason

*TCM* traditional Chinese medicine

### Trial procedure

**Fig. 1:**
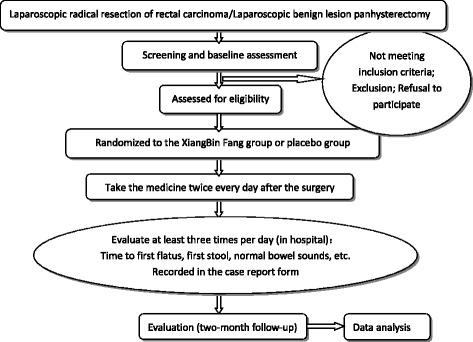
Participant flowchart

**Fig. 2:**
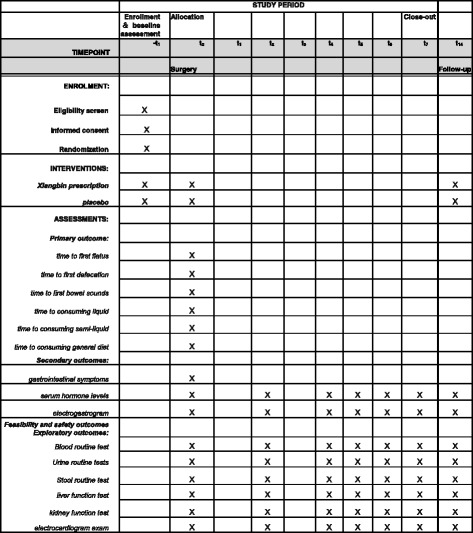
Standard Protocol Items: Recommendations for Interventional Trials (SPIRIT) Figure for Xiangbin prescription for the recovery of gastrointestinal function after abdominal surgery (XBPRS) trial

The entire trial will include a preoperative assessment, an intervention phase (generally 1–7 days), and a 2-month follow-up phase. Included subjects will be randomized to groups receiving XBP or a placebo, using a stratified random system, and acquire a random number (corresponding to numbered drugs) in this phase. Participants will be instructed to take the medicine twice every day after surgery (excluding the surgery day) at 09:00 and 16:00 until defecation occurs. Any remaining medicine will be recovered and recorded. Subjects will be evaluated at least three times per day (between 01:00 and 09:00, 09:00 and 17:00, and 17:00 and 01:00) and the results recorded in a Case Report Form (CRF) until they leave the hospital. The participant flowchart is shown in Fig. 1 and the Standard Protocol Items: Recommendations for Interventional Trials (SPIRIT) Figure for the trial is shown in Fig. 2. Adverse events will be monitored by telephone calls to subjects on the 14th and 60th days after surgery.

### Intervention

The oral Chinese herbal medicine granule (XBP or placebo) is produced by Guangdong Province Engineering Technology Research Institute of TCM, which holds a Good Manufacturing Practice certificate.

After surgery, all included subjects will be administered conventional therapies, including antibiotics and liquid support. Both groups will receive individually packaged doses of XBP or placebo, with instructions that each dose should be dissolved in warm water and consumed per os twice daily after surgery at 09:00 and 16:00 until defecation occurs.

#### Placebo

The placebo is produced by the same manufacturer as the XBP, and consists of starch with no active ingredients. It is dextrin which matched as closely as possible to the appearance and taste of the XBP. The dose and administration instructions for the XBP and placebo will be identical.

### Randomization

A stratified randomization design will be adopted. Random numbers will be generated by the website http://www.gztcmgcp.com/sjxt/login.asp and saved by professional statisticians.

### Double-blinding

Participants and investigators will be blinded. Packaging and labelling of drugs will be conducted by medical staff independent from the research team. Neither the subjects nor the investigators will know the types of drugs taken by each subject. Blind codes will be broken after all processes are completed, or at the request of the Human Research Ethics Committee, or if there is a serious adverse event.

### Quality control

To ensure the quality of the research, this study protocol has undergone multiple modifications and revisions by relevant digestive disease specialists, experienced surgeons, professional statisticians, and research methodologists. To maintain data objectivity, we will ensure that the observers and statisticians are blinded. The whole process will be monitored by independent quality inspectors. The CRF will be completely strictly and in accordance with the CRF instructions; in addition, the original medical history and data recorded in the CRF are not permitted to be altered; any modifications must be explained in detail and accompanied by the signature of the individual who made the change. All laboratory data must be recorded thoroughly, significantly abnormal data checked repeatedly and the physician will be required to make any necessary instructions in response to such information. Since any remaining medicine will be recovered and recorded, we will be able to objectively determine patient compliance.

### Outcome measures

1. Primary outcome measures: time to first flatus, time to first defecation, time to first bowel sounds, time to consuming liquid/semi-liquid/general diet

2. Secondary outcome measures: gastrointestinal symptoms, such as abdominal distention and pain, nausea, vomiting; serum hormone levels, including ghrelin, motilin, corticotropin-releasing hormone, and bradykinin; electrogastrogram

### Safety assessment

The three routine tests (blood, urine, stool), liver and kidney function tests, and an electrocardiogram will be performed both before and after treatment. Adverse events occurring at any time during the treatment or follow-up phases will be observed and recorded in detail.

### Sample size estimation

The study consists of two groups: the sample size estimation was based on our previous prospective clinical study performed in patients who underwent gynecologic transabdominal surgery [1]. Time to first flatus was assumed to be 22.06 and 26.78 h between the XiangBin granules and the placebo groups, respectively. The minimum detectable difference, 4.72 h, was used to estimate the value of the overall parameters. PEMS 3.1 Software for Windows was used to randomly assign the patients to the treatment or the placebo group. The rounded sample size for each group was 89. Assuming a 15% dropout rate, the final sample size would be 105 for each group. Therefore, 420 participants who have undergone laparoscopic radical resection of rectal carcinoma and laparoscopic panhysterectomy of benign lesions will been recruited.

### Data collection and statistical analysis

1. Data will be collected from participants via daily face-to-face and telephone interviews and recorded in the CRF
2. The statistical package, Epidata 3.1, will be used for data entry. Two people will perform data entry independently, and they will be trained in advance and pass a test before they begin the work. The data will then be transferred to Statistical Package for the Social Sciences (SPSS) software for data analysis
3. The statistical analyses were pre-specified and performed on an intention-to-treat (ITT) basis. The ITT analysis will include all patients who were randomly recruited, and the per-protocol (PP) analysis includes patients who completed the study and did not have major protocol violations. All analyses are based on the ITT population, and the result of the ITT analysis is compared with the PP analysis to assess the sensitivity. All missing data are analyzed using the last observation carried forward (LOCF) imputation method. Changes from baseline in outcome measurement among the two groups are analyzed using analysis of variance (ANOVA) or the Kruskal-Wallis test when appropriate. The Bonferroni methods for multi-comparisons are performed if the tests are statistically significant. Safety analysis is analyzed mainly using descriptive statistics. All statistical tests are in a two-tailed t test at a 5% level of significance (Additional file 1).
4. Data will be analyzed by an independent statistician. Data for continuous variables are reported using mean (standard deviation; SD) for normally distributed data or median (range) for skewed data. Data for categorical variables are expressed as number (percentage). Intergroup differences are assessed for significance using Student’s *t* test for normally distributed continuous variables or the Mann-Whitney U test for skewed continuous variables. Intergroup differences in categorical data are assessed using the *χ*^2^ test or Fisher’s exact tests (two-tailed), as appropriate. Length of hospital stay is calculated using Kaplan-Meier analysis to compare differences between groups. Data are analyzed using SPSS 19.0 (IBM, Armonk, NY, USA), with the threshold of significance defined as a two-tailed *p* < 0.05.

## Discussion

In the perioperative period, surgical trauma, effects of anesthesia, the inflammatory response, carbon dioxide pneumoperitoneum, long-term postoperative bed rest, and other factors can cause postoperative gastrointestinal dysfunction in abdominal surgery patients [12] and investigating this problem in patients may be important if it is severe or protracted. The adverse effects of gastrointestinal dysfunction can lead to infection and intestinal adhesions, impeding recovery of function for patients, increasing the medical burden, and prolonging hospitalization. Numerous treatments are used by clinicians to address these issues, including postoperative fasting, early ambulation, enema, chewing gum [13], gastroprokinetic drugs [14], Chinese medicine [15], and acupuncture [16]. This study is designed to evaluate the efficacy and safety of the Chinese medicine, XBP, for the treatment of postoperative gastrointestinal dysfunction in abdominal surgery patients, and to provide a scientific basis for use of this medicine.

1. Basic research into the Chinese medicine, XBP
  XBP is prescribed as a standard prescription for recovery of postoperative gastrointestinal dysfunction after abdominal surgery in our hospital. Preliminary tests [17] have demonstrated that XBP can remarkably improve gastrointestinal motility, and ameliorate the symptoms of dogs after abdominal surgery, and suggested that the treatment may function by mediating the release of endogenous ghrelin and stimulation of the cholinergic neurons of the enteric nervous system. A prospective, clinical [18], randomized, single-blind, placebo-controlled trial conducted in our hospital proved that XBP can effectively promote the recovery of gastrointestinal function after transabdominal gynecological surgery.
2. Features of this study protocol
  (1) the study is designed as a double-blind, randomized controlled, clinical, pivotal trial, which is recognized as the most conclusive research method for therapeutic evaluation; (2) since the majority of outcome measures used to examine impacts on gastrointestinal symptoms remain uncertain, we measured gastrointestinal symptoms by combining primary and secondary outcome measures to ensure scientific objectivity; (3) before patients are enrolled in the study, practitioners will be required to explain the procedure in detail, including the nature, purpose, potential benefits and risks, the rights and obligations of the subject, and the alternative treatments, etc. Patients will only be included in the trial if they are willing to sign an informed consent. Clinicians will provide standard treatment when subjects refuse to participate in the study. The study will strictly adhere to the principles of medical ethics; (4) since the main phase of the protocol will be concluded during hospitalization, practitioners will be able to discover and manage adverse effects immediately, ensuring the security of the study. Practitioners will maintain good communication with subjects by exchanging telephone numbers for use after they leave the hospital.

The study has been developed according to Standard Protocol Items: Recommendations for Interventional Trials (SPIRIT) 2013 [19], the SPIRIT 2013 explanation and elaboration: guidance for protocols of clinical trials [20] (the SPIRIT Checklist is included as Additional file 2) and the Consolidated Standards of Reporting Trials (CONSORT) Statement [21].

### Trail status

Recruitment to this trial is currently ongoing.

## Additional files

Additional file 1: Statistical Analysis Plan. (DOCX 130 kb)

Additional file 2: SPIRIT Checklist for the XBPRS trial protocol. (DOC 150 kb)

## Abbreviations

ANOVA: Analysis of variance
CONSORT: Consolidated Standards of Reporting Trials
CRF: Case Report Form
GCP: Good Clinical Practice
GOP: Ginseng oligopeptides
ITT: Intention-to-treat
LOCF: Last observation carried forward
POI: Postoperative ileus
PP: Per-protocol
SPIRIT: Standard Protocol Items: Recommendations for Interventional Trials
SPSS: Statistical Package for the Social Sciences
TCM: Traditional Chinese medicine
XBP: Xiangbin prescription
XBPRS: Xiangbin prescription for the recovery of gastrointestinal function after abdominal surgery
