# Supplementary material for: Xiangbin prescription for the recovery of gastrointestinal function after abdominal surgery (the XBPRS trial): study protocol for a randomized controlled trial
Source: Trials. 2018 Feb 27;19:146. doi: 10.1186/s13063-018-2484-z (PMC5830341; doi:10.1186/s13063-018-2484-z)
Supplement: Supplementary file 1 — Statistical Analysis Plan. (DOCX 130 kb) [file 13063_2018_2484_MOESM1_ESM.docx]

**中药香槟方组对腹部术后胃肠功能恢复作用的临床研究**

**Chinese herbal medicine (Xiangbin prescription) for recovery of gastrointestinal function after abdominal surgery**

**统计分析计划**

**Statistical Analysis Plan**

**广州中医药大学第二附属医院（广东省中医院）**

**中药香槟方组对腹部术后胃肠功能恢复作用的临床研究课题组**

**2017年6月**

**目 录**

分析目的…………………………………………………………………………3

主要分析内容……………………………………………………………………3

统计分析集………………………………………………………………………3

数据整理与探索性分析…………………………………………………………3

正式的统计分析…………………………………………………………………3

统计分析方法……………………………………………………………………3

完成情况…………………………………………………………………………5

基线分析…………………………………………………………………………6

主要疗效指标分析………………………………………………………………12

其他症状、体征分析……………………………………………………………13

胃肠动力学指标…………………………………………………………………19

胃肠激素类指标…………………………………………………………………20

术后第14天随访-炎性肠梗阻…………………………………………………21

术后第14天随访-术后7天未肛门排便者……………………………………21

不良反应/不良事件…………………………………………………………… 23

安全性实验室指标………………………………………………………………24

合并用药…………………………………………………………………………25

依从性评价………………………………………………………………………25

剔除退出病例分析………………………………………………………………26

分组死亡情况……………………………………………………………………27

患者记录卡………………………………………………………………………27

**1.分析目的：**客观评价以中药香槟方为主的中医口服疗法促进腹部术后胃肠功能恢复的疗效与安全性，完成课题统计分析报告。

**2.主要分析内容：**

（1）分析术前的基线情况。

（2）分析术后中药香槟方干预后，治疗组与对照组主要疗效指标（首次排气时间、首次排便时间、肠鸣音恢复正常时间）的差异。

（3）进行中药香槟方对腹部术后胃肠功能恢复作用的临床安全性分析。

**3.统计分析集：**

共三个数据集：符合方案受试者（per-protocol subjects analysis，PPS）分析集、意向性治疗分析（intention-to-treat analysis，ITT采用接转法）分析集、安全性（safety set）分析集。数据集的分析均在正版SPSS18.0统计分析软件包上实现。

**4.数据整理与探索性分析：**

根据研究目的和疗效相关指标的计算内容，进行数据的审查、核对、整理及探索性分析，了解各数值变量的最大值、最小值，进行相应变量量纲的转换和统计生成新变量。对连续型计量变量的分布进行正态性检验。初步进行组间比较和探索性分析。

**5.正式的统计分析：**

首先是描述性统计分析，列出各数值变量的频数分布、均数（标准差）、min、max及中位数（*M*），然后进行治疗前患者一般情况、手术相关情况及各项观察指标和实验室指标等的基线比较，治疗后主要疗效指标的组间比较，及各症状体征、胃肠动力学指标、胃肠激素类指标等的组间比较。并进行安全性评价、合并用药分析、治疗依从性分析、剔除与退出病例分析等。

**6.统计分析方法：**

**（1）描述性分析：**对各观察时点的各观察指标的计量资料采用均数（标准差）、中位数、最小值和最大值等进行描述性分析；计数资料采用构成比和率进行描述性分析。

**（2）统计推断性分析：**

①计量资料：计算各指标的均数和标准差，进行分布的检验，满足正态分布且方差齐性者，治疗组与安慰剂组间的比较采用方差分析Dunnett法。非正态分布或方差不齐者，采用秩和检验及两两比较（(Kruskal-Wallis H 法及两两比较的Mann-Whitney U 检验），并进行检验水平α的校正；组间基线不齐时，可采用协方差分析。多个观察时点组间比较采用重复测量方差分析等。

②计数资料：计算各指标的构成比及率，组间比较采用$\chi^{2}$检验（或确切概率法）。

③等级资料：组间比较采用秩和检验(Kruskal-Wallis H 法)。

④疗效影响因素的分析：采用非条件logistic回归分析、术后首次排气、排便及肠鸣音恢复正常的中为时间采用采用生存分析（Kaplan-Meier）、COX回归分析等。

⑤正态性检验，检验水平α=0.10。

⑥组间比较α=0.05，两两比较α=0.0125。

**（3）安全性分析：**

各组病例安全性分析主要以描述性统计为主，包括不良事件发生率及不良事件的具体描述；实验室检验结果在试验前后的变化情况；发生异常改变及其与试验用药品的关系等的描述性分析。

**（4）合并用药分析**

主要对各组合并用药情况进行描述，并比较合并用药率是否有差异。

**（4）依从性分析：**

采用描述性分析对各组治疗情况的依从性进行分析。

**（5）脱落病例分析：**

对脱落病例，需说明所有病例脱落的原因，计算脱落病例占总的观察病例的比例，对所有脱落病例基线的临床特征进行分析，并进行组间的描述性分析。

**一、完成情况**

**表1 各中心完成病例情况一览表**

| 中心 | N | 安慰剂组 | | | | 中药香槟方组 | | | |
| --- | --- | --- | --- | --- | --- | --- | --- | --- | --- |
|  |  | 入组 | 剔除 | 脱落 | 完成 | 入组 | 剔除 | 脱落 | 完成 |
| 广州中医药大学第二附属医院 |  |  |  |  |  |  |  |  |  |

**病例入组流程（图）**

**二、基线分析**

**表2 两组治疗前一般资料情况**

| 项目 | | 安慰剂组 | | 中药香槟方组 | | $\chi^{2}$ | *P* |
| --- | --- | --- | --- | --- | --- | --- | --- |
|  |  | *N* | % | *N* | % |  |  |
| 性别 | 男 |  |  |  |  |  |  |
|  | 女 |  |  |  |  |  |  |
| 职业 | 专业技术人员 |  |  |  |  |  |  |
|  | 企事业负责人 |  |  |  |  |  |  |
|  | 办事员 |  |  |  |  |  |  |
|  | 商业工作者 |  |  |  |  |  |  |
|  | 服务人员 |  |  |  |  |  |  |
|  | 农林渔牧业人员 |  |  |  |  |  |  |
|  | 生产运输体力劳动者 |  |  |  |  |  |  |
|  | 其他 |  |  |  |  |  |  |
| 既往腹部手术史 |  |  |  |  |  |  |  |
|  | 无 |  |  |  |  |  |  |
|  | 有 |  |  |  |  |  |  |
| 既往三个月大便性状 | 质硬 |  |  |  |  |  |  |
|  | 质烂 |  |  |  |  |  |  |
|  | 质软 |  |  |  |  |  |  |
|  | 水样便 |  |  |  |  |  |  |
|  | 正常 |  |  |  |  |  |  |
| 心电图 | 正常 |  |  |  |  |  |  |
|  | 异常 |  |  |  |  |  |  |

表2-1 两组治疗前基础疾病情况

| 基础疾病 | | 安慰剂组 | 中药香槟方组 | $\chi^{2}$ | *P* |
| --- | --- | --- | --- | --- | --- |
| 糖尿病 | 有 |  |  |  |  |
|  | 无 |  |  |  |  |
| 冠心病 | 有 |  |  |  |  |
|  | 无 |  |  |  |  |
| 慢性阻塞性肺病 | 有 |  |  |  |  |
|  | 无 |  |  |  |  |
| 高血压 | 有 |  |  |  |  |
|  | 无 |  |  |  |  |
| 其他 | 有 |  |  |  |  |
|  | 无 |  |  |  |  |

表2-2 两组治疗前一般资料情况

| 项目 | | 安慰剂组 | 中药香槟方组 | *$\chi^{2}$/F* | *P* |
| --- | --- | --- | --- | --- | --- |
| 年龄 | n |  |  |  |  |
|  | $\bar{x}\pm s$ |  |  |  |  |
|  | *M* |  |  |  |  |
|  | Min |  |  |  |  |
|  | Max |  |  |  |  |
| 既往三个月大便习惯（次/天） | n |  |  |  |  |
|  | $\bar{x}\pm s$ |  |  |  |  |
|  | M |  |  |  |  |
|  | Min |  |  |  |  |
|  | Max |  |  |  |  |

表3 两组治疗前手术相关情况

| 项目 | | 安慰剂组 | | 中药香槟方组 | | $\chi^{2}$ | *P* |
| --- | --- | --- | --- | --- | --- | --- | --- |
|  |  | *N* | % | *N* | % |  |  |
| 手术分类 | 急诊手术 |  |  |  |  |  |  |
|  | 择期手术 |  |  |  |  |  |  |
|  | 限期手术 |  |  |  |  |  |  |
| 术前饮食 | 半流 |  |  |  |  |  |  |
|  | 全流 |  |  |  |  |  |  |
|  | 禁食 |  |  |  |  |  |  |
| 泻药 | 无 |  |  |  |  |  |  |
|  | 有 |  |  |  |  |  |  |
| 灌肠 | 无 |  |  |  |  |  |  |
|  | 有 |  |  |  |  |  |  |
| 胃肠道手术 | 否 |  |  |  |  |  |  |
|  | 是 |  |  |  |  |  |  |
| 麻醉方式 | 全麻 |  |  |  |  |  |  |
|  | 硬膜外麻醉 |  |  |  |  |  |  |
|  | 腰硬联合麻醉 |  |  |  |  |  |  |
|  | 全麻+硬膜外麻醉 |  |  |  |  |  |  |
| 术中是否冲洗 | 否 |  |  |  |  |  |  |
|  | 是 |  |  |  |  |  |  |
| 术后是否放置腹腔引流 | 否 |  |  |  |  |  |  |
|  | 是 |  |  |  |  |  |  |
| 术后是否放置尿管 | 否 |  |  |  |  |  |  |
|  | 是 |  |  |  |  |  |  |
| 术后是否放置胃管 | 否 |  |  |  |  |  |  |
|  | 是 |  |  |  |  |  |  |
|  | 否 |  |  |  |  |  |  |
|  | 是 |  |  |  |  |  |  |

**表3-1 两组治疗前手术相关情况**

| 项目 | | 安慰剂组 | 中药香槟方组 | *$\chi^{2}$/F* | *P* |
| --- | --- | --- | --- | --- | --- |
| 麻醉时间 | n |  |  |  |  |
|  | $\bar{x}\pm s$ |  |  |  |  |
|  | *M* |  |  |  |  |
|  | Min |  |  |  |  |
|  | Max |  |  |  |  |
|  | $\bar{x}\pm s$ |  |  |  |  |
|  | *M* |  |  |  |  |
|  | Min |  |  |  |  |
|  | Max |  |  |  |  |
| 出血量估计  （毫升） | n |  |  |  |  |
|  | $\bar{x}\pm s$ |  |  |  |  |
|  | *M* |  |  |  |  |
|  | Min |  |  |  |  |
|  | Max |  |  |  |  |

**表4 两组术后第一天治疗前各项指标**

| 项目 | | 安慰剂组 | | 中药香槟方组 | | $\chi^{2}$ | *P* |
| --- | --- | --- | --- | --- | --- | --- | --- |
|  |  | *N* | % | *N* | % |  |  |
| 肛门排气 |  |  |  |  |  |  |  |
|  | 无 |  |  |  |  |  |  |
|  | 有 |  |  |  |  |  |  |
| 肛门排便 |  |  |  |  |  |  |  |
|  | 无 |  |  |  |  |  |  |
|  | 有 |  |  |  |  |  |  |
| 腹胀 | 无 |  |  |  |  |  |  |
|  | 较明显，可忍受 |  |  |  |  |  |  |
|  | 全腹胀满，难忍受 |  |  |  |  |  |  |
| 腹痛 | 无 |  |  |  |  |  |  |
|  | 有，但无需治疗 |  |  |  |  |  |  |
|  | 有，需要治疗 |  |  |  |  |  |  |
| 恶心呕吐 | 无 |  |  |  |  |  |  |
|  | 欲呕而未吐 |  |  |  |  |  |  |
|  | 呕吐胃液或胆汁 |  |  |  |  |  |  |
| 饥饿感 | 无 |  |  |  |  |  |  |
|  | 一般 |  |  |  |  |  |  |
|  | 明显 |  |  |  |  |  |  |
| 体温 |  |  |  |  |  |  |  |
|  | $\leq$ 37.5 |  |  |  |  |  |  |
|  | 37.5-39 |  |  |  |  |  |  |
|  | $\geq$ 39度 |  |  |  |  |  |  |
| 肠鸣音 |  |  |  |  |  |  |  |
| 7.00-9.00AM | 正常 |  |  |  |  |  |  |
|  | 减弱 |  |  |  |  |  |  |
|  | 无 |  |  |  |  |  |  |
| 11.00-13.00 | 正常 |  |  |  |  |  |  |
|  | 减弱 |  |  |  |  |  |  |
|  | 无 |  |  |  |  |  |  |
| 15.00-17.00 | 正常 |  |  |  |  |  |  |
|  | 减弱 |  |  |  |  |  |  |
|  | 无 |  |  |  |  |  |  |
| 19.00-21.00 | 正常 |  |  |  |  |  |  |
|  | 减弱 |  |  |  |  |  |  |
|  | 无 |  |  |  |  |  |  |
| 23.00-1.00 | 正常 |  |  |  |  |  |  |
|  | 减弱 |  |  |  |  |  |  |
|  | 无 |  |  |  |  |  |  |
| 3.00-5.00 | 正常 |  |  |  |  |  |  |
|  | 减弱 |  |  |  |  |  |  |
|  | 无 |  |  |  |  |  |  |
| 并发症 | 无 |  |  |  |  |  |  |
|  | 有 |  |  |  |  |  |  |

**表5 两组术后第一天治疗前实验室指标**$\bar{x}\pm s$

| 项目 | | 安慰剂组 | | 中药香槟方组 | | *F*/$\chi^{2}$ | *P* |
| --- | --- | --- | --- | --- | --- | --- | --- |
|  |  | $\bar{x}\pm s$ | *M* | $\bar{x}\pm s$ | *M* |  |  |
| 血常规 | n |  |  |  |  |  |  |
|  | （RBC） |  |  |  |  |  |  |
|  | (Hb) |  |  |  |  |  |  |
|  | (WBC) |  |  |  |  |  |  |
|  | (PLT) |  |  |  |  |  |  |
| 尿常规 | n |  |  |  |  |  |  |
|  | 尿蛋白 |  |  |  |  |  |  |
|  | 白细胞 |  |  |  |  |  |  |
|  | 红细胞 |  |  |  |  |  |  |
| 血生化 | n |  |  |  |  |  |  |
|  | （ALT） |  |  |  |  |  |  |
|  | （BUN） |  |  |  |  |  |  |
|  | （Cr） |  |  |  |  |  |  |
|  | K+ |  |  |  |  |  |  |
|  | Na+ |  |  |  |  |  |  |
|  | Cl- |  |  |  |  |  |  |

**表5-1 两组术后第一天治疗前舌脉情况**

| 舌脉 | 安慰剂组 | 中药香槟方组 | $\chi^{2}$ | *P* |
| --- | --- | --- | --- | --- |
| **舌质：**淡红 |  |  |  |  |
| 暗红 |  |  |  |  |
| 红绛 |  |  |  |  |
| 其他 |  |  |  |  |
| **舌苔：**薄 |  |  |  |  |
| 厚 |  |  |  |  |
| 白 |  |  |  |  |
| 黄 |  |  |  |  |
| 黑 |  |  |  |  |
| 腻 |  |  |  |  |
| 无苔 |  |  |  |  |
| 少苔 |  |  |  |  |
| 剥苔 |  |  |  |  |
| 其他 |  |  |  |  |
| **脉象：**弦 |  |  |  |  |
| 滑 |  |  |  |  |
| 沉 |  |  |  |  |
| 数 |  |  |  |  |
| 缓 |  |  |  |  |
| 细 |  |  |  |  |
| 其他 |  |  |  |  |

**三、主要疗效指标分析**

治疗组均与安慰剂组进行比较，采用方差分析（Dunnett’s test）。

**表6 两组患者术后首次排气、首次排便和肠鸣音正常时间**$\bar{x}\pm s$,*M***（ITT分析）**

| 项目 | 安慰剂组 | | 中药香槟方组 | | *F/$\chi^{2}$* | *P* |
| --- | --- | --- | --- | --- | --- | --- |
|  | $\bar{x}\pm s$ | *M* | $\bar{x}\pm s$ | *M* |  |  |
| 首次排气时间 |  |  |  |  |  |  |
| 首次排便时间 |  |  |  |  |  |  |
| 肠鸣音正常时间 |  |  |  |  |  |  |

**表7 两组患者术后首次排气、首次排便和肠鸣音正常时间**$\bar{x}\pm s$,*M***（PP分析）**

| 观察指标 | 安慰剂组 | | 中药香槟方组 | | *F/$\chi^{2}$* | *P* |
| --- | --- | --- | --- | --- | --- | --- |
|  | $\bar{x}\pm s$ | *M* | $\bar{x}\pm s$ | *M* |  |  |
| 首次排气时间 |  |  |  |  |  |  |
| 首次排便时间 |  |  |  |  |  |  |
| 肠鸣音正常时间 |  |  |  |  |  |  |

**四．其他症状、体征分析**

秩和检验

时点1：术后第1天第1次治疗前；时点2：术后第2天第2次治疗前；时点3：术后第3天第3次治疗前；时点4：术后第4天第4次治疗前；时点5：术后第5天第5次治疗前；时点6：术后第6天第6次治疗前；时点7：术后第7天

**表8 两组术后各时点腹胀情况（ITT分析）**

| 观察时点 | | 安慰剂组 | | 中药香槟方组 | | *$\chi^{2}$* | *P* |
| --- | --- | --- | --- | --- | --- | --- | --- |
|  |  | *N* | % | *N* | % |  |  |
| 时点1 | 0分 |  |  |  |  |  |  |
|  | 2分 |  |  |  |  |  |  |
|  | 4分 |  |  |  |  |  |  |
| 时点2 | 0分 |  |  |  |  |  |  |
|  | 2分 |  |  |  |  |  |  |
|  | 4分 |  |  |  |  |  |  |
| 时点3 | 0分 |  |  |  |  |  |  |
|  | 2分 |  |  |  |  |  |  |
|  | 4分 |  |  |  |  |  |  |
| 时点4 | 0分 |  |  |  |  |  |  |
|  | 2分 |  |  |  |  |  |  |
|  | 4分 |  |  |  |  |  |  |
| 时点5 | 0分 |  |  |  |  |  |  |
|  | 2分 |  |  |  |  |  |  |
|  | 4分 |  |  |  |  |  |  |
| 时点6 | 0分 |  |  |  |  |  |  |
|  | 2分 |  |  |  |  |  |  |
|  | 4分 |  |  |  |  |  |  |
| 时点7 | 0分 |  |  |  |  |  |  |
|  | 2分 |  |  |  |  |  |  |
|  | 4分 |  |  |  |  |  |  |

0分：无腹胀；2分：较明显，尚可忍受；4分：全腹胀满，较难忍受，可见肠形

**表9 两组术后各时点腹胀情况（PP分析）**

| 观察时点 | | 安慰剂组 | | 中药香槟方组 | | *$\chi^{2}$* | *P* |
| --- | --- | --- | --- | --- | --- | --- | --- |
|  |  | *N* | % | *N* | % |  |  |
| 时点1 | 0分 |  |  |  |  |  |  |
|  | 2分 |  |  |  |  |  |  |
|  | 4分 |  |  |  |  |  |  |
| 时点2 | 0分 |  |  |  |  |  |  |
|  | 2分 |  |  |  |  |  |  |
|  | 4分 |  |  |  |  |  |  |
| 时点3 | 0分 |  |  |  |  |  |  |
|  | 2分 |  |  |  |  |  |  |
|  | 4分 |  |  |  |  |  |  |
| 时点4 | 0分 |  |  |  |  |  |  |
|  | 2分 |  |  |  |  |  |  |
|  | 4分 |  |  |  |  |  |  |
| 时点5 | 0分 |  |  |  |  |  |  |
|  | 2分 |  |  |  |  |  |  |
|  | 4分 |  |  |  |  |  |  |
| 时点6 | 0分 |  |  |  |  |  |  |
|  | 2分 |  |  |  |  |  |  |
|  | 4分 |  |  |  |  |  |  |
| 时点7 | 0分 |  |  |  |  |  |  |
|  | 2分 |  |  |  |  |  |  |
|  | 4分 |  |  |  |  |  |  |

0分：无腹胀；2分：较明显，尚可忍受；4分：全腹胀满，较难忍受，可见肠形

**表10 两组术后各时点腹痛情况（ITT分析）**

| 观察时点 | | 安慰剂组 | | 中药香槟方组 | | *$\chi^{2}$* | *P* |
| --- | --- | --- | --- | --- | --- | --- | --- |
|  |  | *N* | % | *N* | % |  |  |
| 时点1 | 0分 |  |  |  |  |  |  |
|  | 2分 |  |  |  |  |  |  |
|  | 4分 |  |  |  |  |  |  |
| 时点2 | 0分 |  |  |  |  |  |  |
|  | 2分 |  |  |  |  |  |  |
|  | 4分 |  |  |  |  |  |  |
| 时点3 | 0分 |  |  |  |  |  |  |
|  | 2分 |  |  |  |  |  |  |
|  | 4分 |  |  |  |  |  |  |
| 时点4 | 0分 |  |  |  |  |  |  |
|  | 2分 |  |  |  |  |  |  |
|  | 4分 |  |  |  |  |  |  |
| 时点5 | 0分 |  |  |  |  |  |  |
|  | 2分 |  |  |  |  |  |  |
|  | 4分 |  |  |  |  |  |  |
| 时点6 | 0分 |  |  |  |  |  |  |
|  | 2分 |  |  |  |  |  |  |
|  | 4分 |  |  |  |  |  |  |
| 时点7 | 0分 |  |  |  |  |  |  |
|  | 2分 |  |  |  |  |  |  |
|  | 4分 |  |  |  |  |  |  |

0分：无腹痛；2分：有疼痛，但无需止痛治疗；4分：有疼痛，需止痛治疗

**表11 两组术后各时点腹痛情况（PP分析）**

| 观察时点 | | 安慰剂组 | | 中药香槟方组 | | *$\chi^{2}$* | *P* |
| --- | --- | --- | --- | --- | --- | --- | --- |
|  |  | *N* | % | *N* | % |  |  |
| 时点1 | 0分 |  |  |  |  |  |  |
|  | 2分 |  |  |  |  |  |  |
|  | 4分 |  |  |  |  |  |  |
| 时点2 | 0分 |  |  |  |  |  |  |
|  | 2分 |  |  |  |  |  |  |
|  | 4分 |  |  |  |  |  |  |
| 时点3 | 0分 |  |  |  |  |  |  |
|  | 2分 |  |  |  |  |  |  |
|  | 4分 |  |  |  |  |  |  |
| 时点4 | 0分 |  |  |  |  |  |  |
|  | 2分 |  |  |  |  |  |  |
|  | 4分 |  |  |  |  |  |  |
| 时点5 | 0分 |  |  |  |  |  |  |
|  | 2分 |  |  |  |  |  |  |
|  | 4分 |  |  |  |  |  |  |
| 时点6 | 0分 |  |  |  |  |  |  |
|  | 2分 |  |  |  |  |  |  |
|  | 4分 |  |  |  |  |  |  |
| 时点7 | 0分 |  |  |  |  |  |  |
|  | 2分 |  |  |  |  |  |  |
|  | 4分 |  |  |  |  |  |  |

0分：无腹痛；2分：有疼痛，但无需止痛治疗；4分：有疼痛，需止痛治疗

**表12 两组术后各时点恶心呕吐情况（ITT分析）**

| 观察时点 | | 安慰剂组 | | 中药香槟方组 | | *$\chi^{2}$* | *P* |
| --- | --- | --- | --- | --- | --- | --- | --- |
|  |  | *N* | % | *N* | % |  |  |
| 时点1 | 0分 |  |  |  |  |  |  |
|  | 2分 |  |  |  |  |  |  |
|  | 4分 |  |  |  |  |  |  |
| 时点2 | 0分 |  |  |  |  |  |  |
|  | 2分 |  |  |  |  |  |  |
|  | 4分 |  |  |  |  |  |  |
| 时点3 | 0分 |  |  |  |  |  |  |
|  | 2分 |  |  |  |  |  |  |
|  | 4分 |  |  |  |  |  |  |
| 时点4 | 0分 |  |  |  |  |  |  |
|  | 2分 |  |  |  |  |  |  |
|  | 4分 |  |  |  |  |  |  |
| 时点5 | 0分 |  |  |  |  |  |  |
|  | 2分 |  |  |  |  |  |  |
|  | 4分 |  |  |  |  |  |  |
| 时点6 | 0分 |  |  |  |  |  |  |
|  | 2分 |  |  |  |  |  |  |
|  | 4分 |  |  |  |  |  |  |
| 时点7 | 0分 |  |  |  |  |  |  |
|  | 2分 |  |  |  |  |  |  |
|  | 4分 |  |  |  |  |  |  |

0分：无恶心呕吐；2分：恶心欲呕而未吐；4分：呕吐胃液或胆汁

**表13 两组术后各时点恶心呕吐情况（PP分析）**

| 观察时点 | | 安慰剂组 | | 中药香槟方组 | | *$\chi^{2}$* | *P* |
| --- | --- | --- | --- | --- | --- | --- | --- |
|  |  | *N* | % | *N* | % |  |  |
| 时点1 | 0分 |  |  |  |  |  |  |
|  | 2分 |  |  |  |  |  |  |
|  | 4分 |  |  |  |  |  |  |
| 时点2 | 0分 |  |  |  |  |  |  |
|  | 2分 |  |  |  |  |  |  |
|  | 4分 |  |  |  |  |  |  |
| 时点3 | 0分 |  |  |  |  |  |  |
|  | 2分 |  |  |  |  |  |  |
|  | 4分 |  |  |  |  |  |  |
| 时点4 | 0分 |  |  |  |  |  |  |
|  | 2分 |  |  |  |  |  |  |
|  | 4分 |  |  |  |  |  |  |
| 时点5 | 0分 |  |  |  |  |  |  |
|  | 2分 |  |  |  |  |  |  |
|  | 4分 |  |  |  |  |  |  |
| 时点6 | 0分 |  |  |  |  |  |  |
|  | 2分 |  |  |  |  |  |  |
|  | 4分 |  |  |  |  |  |  |
| 时点7 | 0分 |  |  |  |  |  |  |
|  | 2分 |  |  |  |  |  |  |
|  | 4分 |  |  |  |  |  |  |

0分：无恶心呕吐；2分：恶心欲呕而未吐；4分：呕吐胃液或胆汁

**表14 两组术后各时点饥饿感情况（ITT分析）**

| 观察时点 | | 安慰剂组 | | 中药香槟方组 | | *$\chi^{2}$* | *P* |
| --- | --- | --- | --- | --- | --- | --- | --- |
|  |  | *N* | % | *N* | % |  |  |
| 时点1 | 0分 |  |  |  |  |  |  |
|  | 2分 |  |  |  |  |  |  |
|  | 4分 |  |  |  |  |  |  |
| 时点2 | 0分 |  |  |  |  |  |  |
|  | 2分 |  |  |  |  |  |  |
|  | 4分 |  |  |  |  |  |  |
| 时点3 | 0分 |  |  |  |  |  |  |
|  | 2分 |  |  |  |  |  |  |
|  | 4分 |  |  |  |  |  |  |
| 时点4 | 0分 |  |  |  |  |  |  |
|  | 2分 |  |  |  |  |  |  |
|  | 4分 |  |  |  |  |  |  |
| 时点5 | 0分 |  |  |  |  |  |  |
|  | 2分 |  |  |  |  |  |  |
|  | 4分 |  |  |  |  |  |  |
| 时点6 | 0分 |  |  |  |  |  |  |
|  | 2分 |  |  |  |  |  |  |
|  | 4分 |  |  |  |  |  |  |
| 时点7 | 0分 |  |  |  |  |  |  |
|  | 2分 |  |  |  |  |  |  |
|  | 4分 |  |  |  |  |  |  |

0分：无饥饿感；2分：一般，想吃一些食物；4分：明显，都想吃

**表15 两组术后各时点饥饿感情况（PP分析）**

| 观察时点 | | 安慰剂组 | | 中药香槟方组 | | *$\chi^{2}$* | *P* |
| --- | --- | --- | --- | --- | --- | --- | --- |
|  |  | *N* | % | *N* | % |  |  |
| 时点1 | 0分 |  |  |  |  |  |  |
|  | 2分 |  |  |  |  |  |  |
|  | 4分 |  |  |  |  |  |  |
| 时点2 | 0分 |  |  |  |  |  |  |
|  | 2分 |  |  |  |  |  |  |
|  | 4分 |  |  |  |  |  |  |
| 时点3 | 0分 |  |  |  |  |  |  |
|  | 2分 |  |  |  |  |  |  |
|  | 4分 |  |  |  |  |  |  |
| 时点4 | 0分 |  |  |  |  |  |  |
|  | 2分 |  |  |  |  |  |  |
|  | 4分 |  |  |  |  |  |  |
| 时点5 | 0分 |  |  |  |  |  |  |
|  | 2分 |  |  |  |  |  |  |
|  | 4分 |  |  |  |  |  |  |
| 时点6 | 0分 |  |  |  |  |  |  |
|  | 2分 |  |  |  |  |  |  |
|  | 4分 |  |  |  |  |  |  |
| 时点7 | 0分 |  |  |  |  |  |  |
|  | 2分 |  |  |  |  |  |  |
|  | 4分 |  |  |  |  |  |  |

0分：无饥饿感；2分：一般，想吃一些食物；4分：明显，都想吃

**五．胃肠动力学指标**

需要临床指导（是否需要餐前和餐后比较？不同时点比较？）

**表16 两组不同时点多导胃肠电图胃体平均幅值**$\bar{x}\pm s$,*M*

| 观察时间 | | 安慰剂组 | | 中药香槟方组 | | *F* | *P* |
| --- | --- | --- | --- | --- | --- | --- | --- |
|  |  | $\bar{x}\pm s$ | *M* | $\bar{x}\pm s$ | *M* |  |  |
| 术前1周 | 餐前 |  |  |  |  |  |  |
|  | 餐后 |  |  |  |  |  |  |
| 术后第  1天 | 餐前 |  |  |  |  |  |  |
|  | 餐后 |  |  |  |  |  |  |
| 术后第  3天 | 餐前 |  |  |  |  |  |  |
|  | 餐后 |  |  |  |  |  |  |
| 术后治疗  结束后 | 餐前 |  |  |  |  |  |  |
|  | 餐后 |  |  |  |  |  |  |

**表17 两组不同时点多导胃肠电图小弯平均幅值**$\bar{x}\pm s$,*M*

| 观察时间 | | 安慰剂组 | | 中药香槟方组 | | *F* | *P* |
| --- | --- | --- | --- | --- | --- | --- | --- |
|  |  | $\bar{x}\pm s$ | *M* | $\bar{x}\pm s$ | *M* |  |  |
| 术前一周 | 餐前 |  |  |  |  |  |  |
|  | 餐后 |  |  |  |  |  |  |
| 术后第1天 | 餐前 |  |  |  |  |  |  |
|  | 餐后 |  |  |  |  |  |  |
| 术后第3天 | 餐前 |  |  |  |  |  |  |
|  | 餐后 |  |  |  |  |  |  |
| 术后治疗结束后 | 餐前 |  |  |  |  |  |  |
|  | 餐后 |  |  |  |  |  |  |

**……**

**……8个部位，11个指标，共88个表**

**表18（序号，以后改） 两组不同时点多导胃肠电图直肠餐前/餐后功率比**$\bar{x}\pm s$,*M*

| 观察时间 | | 安慰剂组 | | 中药香槟方组 | | *F* | *P* |
| --- | --- | --- | --- | --- | --- | --- | --- |
|  |  | $\bar{x}\pm s$ | *M* | $\bar{x}\pm s$ | *M* |  |  |
| 术前一周 | 餐前 |  |  |  |  |  |  |
|  | 餐后 |  |  |  |  |  |  |
| 术后第1天 | 餐前 |  |  |  |  |  |  |
|  | 餐后 |  |  |  |  |  |  |
| 术后第3天 | 餐前 |  |  |  |  |  |  |
|  | 餐后 |  |  |  |  |  |  |
| 术后治疗结束后 | 餐前 |  |  |  |  |  |  |
|  | 餐后 |  |  |  |  |  |  |

**六、胃肠激素类指标**

**表19 两组不同时点胃动素（ng/L）**$\bar{x}\pm s$,*M*

| 观察时点 | 安慰剂组 | | 中药香槟方组 | | *F* | *P* |
| --- | --- | --- | --- | --- | --- | --- |
|  | $\bar{x}\pm s$ | *M* | $\bar{x}\pm s$ | *M* |  |  |
| 术前一周 |  |  |  |  |  |  |
| 术后第1天 |  |  |  |  |  |  |
| 术后第3天 |  |  |  |  |  |  |
| 术后治疗结束后 |  |  |  |  |  |  |

**表20 两组不同时点血管活性肠肽（ng/L）**$\bar{x}\pm s$,*M*

| 观察时点 | 安慰剂组 | | 中药香槟方组 | | *F* | *P* |
| --- | --- | --- | --- | --- | --- | --- |
|  | $\bar{x}\pm s$ | *M* | $\bar{x}\pm s$ | *M* |  |  |
| 术前一周 |  |  |  |  |  |  |
| 术后第1天 |  |  |  |  |  |  |
| 术后第3天 |  |  |  |  |  |  |
| 术后治疗结束后 |  |  |  |  |  |  |

**表21 两组不同时点一氧化氮（ng/L）**$\bar{x}\pm s$,*M*

| 观察时点 | 安慰剂组 | | 中药香槟方组组 | | *F* | *P* |
| --- | --- | --- | --- | --- | --- | --- |
|  | $\bar{x}\pm s$ | *M* | $\bar{x}\pm s$ | *M* |  |  |
| 术前一周 |  |  |  |  |  |  |
| 术后第1天 |  |  |  |  |  |  |
| 术后第3天 |  |  |  |  |  |  |
| 术后治疗结束后 |  |  |  |  |  |  |

**七、术后第14天随访-炎性肠梗阻**

**表22 两组术后第14天炎性肠梗阻发生情况**

| 观察时点 | | 安慰剂组 | | 中药香槟方组 | | $\chi^{2}$ | *P* |
| --- | --- | --- | --- | --- | --- | --- | --- |
|  |  | *N* | % | *N* | % |  |  |
| 肠梗阻 | 否 |  |  |  |  |  |  |
|  | 是 |  |  |  |  |  |  |
| 腹胀 | 否 |  |  |  |  |  |  |
|  | 是 |  |  |  |  |  |  |
| 腹痛 | 否 |  |  |  |  |  |  |
|  | 是 |  |  |  |  |  |  |
| 恶心 | 否 |  |  |  |  |  |  |
|  | 是 |  |  |  |  |  |  |
| 呕吐 | 否 |  |  |  |  |  |  |
|  | 是 |  |  |  |  |  |  |
| 肠鸣音减弱 | 否 |  |  |  |  |  |  |
|  | 是 |  |  |  |  |  |  |
| 肠鸣音消失 | 否 |  |  |  |  |  |  |
|  | 是 |  |  |  |  |  |  |
| 腹平片可见液平 | 否 |  |  |  |  |  |  |
|  | 是 |  |  |  |  |  |  |
| 肠管扩张 | 否 |  |  |  |  |  |  |
|  | 是 |  |  |  |  |  |  |
| 其他 | 否 |  |  |  |  |  |  |
|  | 是 |  |  |  |  |  |  |

**八、术后第14天随访-术后7天未肛门排便者**

**表23 两组术后7天未排便者在术后第14天肛门排便情况**

| 排便情况 | 安慰剂组 | | 中药香槟方组 | | $\chi^{2}$ | *P* |
| --- | --- | --- | --- | --- | --- | --- |
|  | *N* | % | *N* | % |  |  |
| 未排便 |  |  |  |  |  |  |
| 已排便 |  |  |  |  |  |  |

**表24 两组术后7天未排便者在术后第14天肛门排便情况**

| 排便情况 | 安慰剂组 | | 中药香槟方组 | | *F*/$\chi^{2}$ | *P* |
| --- | --- | --- | --- | --- | --- | --- |
|  | $\bar{x}\pm s$ | *M* | $\bar{x}\pm s$ | *M* |  |  |
| 第1次排便时间 |  |  |  |  |  |  |
| 第2次排便时间 |  |  |  |  |  |  |

**表25 两组手术疗后各观察时点舌脉情况**

| 观察时点 | 舌脉 | 安慰剂组 | 中药香槟方组 | $\chi^{2}$ | *P* |
| --- | --- | --- | --- | --- | --- |
| **疗后1天** | **舌质：**淡红 |  |  |  |  |
|  | 暗红 |  |  |  |  |
|  | 红绛 |  |  |  |  |
|  | 其他 |  |  |  |  |
|  | **舌苔：**薄 |  |  |  |  |
|  | 厚 |  |  |  |  |
|  | 白 |  |  |  |  |
|  | 黄 |  |  |  |  |
|  | 黑 |  |  |  |  |
|  | 腻 |  |  |  |  |
|  | 无苔 |  |  |  |  |
|  | 少苔 |  |  |  |  |
|  | 剥苔 |  |  |  |  |
|  | 其他 |  |  |  |  |
|  | **脉象：**弦 |  |  |  |  |
|  | 滑 |  |  |  |  |
|  | 沉 |  |  |  |  |
|  | 数 |  |  |  |  |
|  | 缓 |  |  |  |  |
|  | 细 |  |  |  |  |
|  | 其他 |  |  |  |  |
| **疗后2天** | **舌质：**淡红 |  |  |  |  |
|  | 暗红 |  |  |  |  |
|  | 红绛 |  |  |  |  |
|  | 其他 |  |  |  |  |
|  | **舌苔：**薄 |  |  |  |  |
|  | 厚 |  |  |  |  |
|  | 白 |  |  |  |  |
|  | 黄 |  |  |  |  |
|  | 黑 |  |  |  |  |
|  | 腻 |  |  |  |  |
|  | 无苔 |  |  |  |  |
|  | 少苔 |  |  |  |  |
|  | 剥苔 |  |  |  |  |
|  | 其他 |  |  |  |  |
|  | **脉象：**弦 |  |  |  |  |
|  | 滑 |  |  |  |  |
|  | 沉 |  |  |  |  |
|  | 数 |  |  |  |  |
|  | 缓 |  |  |  |  |
|  | 细 |  |  |  |  |
|  | 其他 |  |  |  |  |
| ：  ：  ：  ： | ：  ：  ：  ： |  |  |  |  |
|  |  |  |  |  |  |
|  |  |  |  |  |  |
|  |  |  |  |  |  |
| **疗后7天** | **舌质：**淡红 |  |  |  |  |
|  | 暗红 |  |  |  |  |
|  | 红绛 |  |  |  |  |
|  | 其他 |  |  |  |  |
|  | **舌苔：**薄 |  |  |  |  |
|  | 厚 |  |  |  |  |
|  | 白 |  |  |  |  |
|  | 黄 |  |  |  |  |
|  | 黑 |  |  |  |  |
|  | 腻 |  |  |  |  |
|  | 无苔 |  |  |  |  |
|  | 少苔 |  |  |  |  |
|  | 剥苔 |  |  |  |  |
|  | 其他 |  |  |  |  |
|  | **脉象：**弦 |  |  |  |  |
|  | 滑 |  |  |  |  |
|  | 沉 |  |  |  |  |
|  | 数 |  |  |  |  |
|  | 缓 |  |  |  |  |
|  | 细 |  |  |  |  |
|  | 其他 |  |  |  |  |

**九、不良反应/不良事件**

**表26 不良反应/不良事件发生例次分析一览表**

| 项目 | 安慰剂组 | 中药香槟方组 | 合计 |
| --- | --- | --- | --- |
| 腹泻？ |  |  |  |
| 胃痛 |  |  |  |
| 恶心 |  |  |  |
| 厌食 |  |  |  |
| 口腔溃疡 |  |  |  |
| 大便隐血 |  |  |  |
| 胃出血 |  |  |  |
| 血液系统小计 |  |  |  |
| 肝功能小计 |  |  |  |
| 肾功能小计 |  |  |  |
| 其他小计 |  |  |  |
| 合计 |  |  |  |

**安全性实验室指标：RBC, Hb, WBC, PLT, ALT, BUN, Cr,心电图等**

**表27 两组血、尿常规、肝肾功能及心电图异常反应发生情况**

| 项目 | 项目 | 安慰剂组 | 中药香槟方组 | 合计 |
| --- | --- | --- | --- | --- |
| 血常规 | 红细胞计数（RBC） |  |  |  |
|  | 血红蛋白(Hb) |  |  |  |
|  | 白细胞计数(WBC) |  |  |  |
|  | 血小板计数(PLT) |  |  |  |
| 尿常规 | 尿蛋白 |  |  |  |
|  | 白细胞 |  |  |  |
|  | 红细胞 |  |  |  |
| 肝肾功能 | 谷丙转氨酶（ALT） |  |  |  |
|  | 尿素氮（BUN） |  |  |  |
|  | 肌酐（Cr） |  |  |  |
|  | 电解质K+ |  |  |  |
|  | 电解质Na+ |  |  |  |
|  | 电解质Cl- |  |  |  |
| 心电图 |  |  |  |  |
| 合计 |  |  |  |  |

**十、合并用药**

中药香槟方组？例，安慰剂组？例，两组差异？统计学意义。

表28 治疗过程中合并用药人次分类情况

| 分组 | 合并中药 | 合并西药 |
| --- | --- | --- |
| 中药香槟方组（n=） |  |  |
| 安慰剂组 |  |  |

**十一、依从性评价（不需进行空白组）**

**表29 依从性情况（%）**

| 依从性(%) | 0 | 10 | 20 | 30 | 40 | 50 | 60 | 70 | 80 | 90 | 100 | 合计 | *Z* | *P* |
| --- | --- | --- | --- | --- | --- | --- | --- | --- | --- | --- | --- | --- | --- | --- |
| 中药香槟方组 |  |  |  |  |  |  |  |  |  |  |  |  |  |  |

依从性分析:依从性按＜80%、80%-120%、＞120%分类，分别计算两组总依从性中各类所占的比例，并采用$\chi^{2}$检验或精确概率法进行比较。

**参照SOP, 中药香槟方组每天2次。治疗时间为9AM、16PM，6天为一个疗程。**

**表30 治疗依从性分析**

| 治疗时间 | | 中药香槟方组 | |
| --- | --- | --- | --- |
|  |  | *N* | % |
| 术后第1天 | >30min |  |  |
|  | =30min |  |  |
|  | <30min |  |  |
|  | 未使用 |  |  |
| 术后第2天 | >30min |  |  |
|  | =30min |  |  |
|  | <30min |  |  |
|  | 未使用 |  |  |
| 术后第3天 | >30min |  |  |
|  | =30min |  |  |
|  | <30min |  |  |
|  | 未使用 |  |  |
| 术后第4天 | >30min |  |  |
|  | =30min |  |  |
|  | <30min |  |  |
|  | 未使用 |  |  |
| 术后第5天 | >30min |  |  |
|  | =30min |  |  |
|  | <30min |  |  |
|  | 未使用 |  |  |
| 术后第6天 | >30min |  |  |
|  | =30min |  |  |
|  | <30min |  |  |
|  | 未使用 |  |  |

**十二、剔除退出病例分析**

**剔除病例分析**

**脱落及中止病例分析：**

共脱落病例？例，其中中药香槟方组？例，安慰剂组？例。脱落原因分析：

表31 各时点脱落/中止不同原因分析

| 脱落  时间 | 疗效指标 | 分组 | 不良事件  /反应 | 出院 | 转院 | 医师决定停止治疗 | 受试者  自行退出 | 其他 |
| --- | --- | --- | --- | --- | --- | --- | --- | --- |
| 时间1 |  | 安慰剂组 |  |  |  |  |  |  |
|  |  | 中药香槟方组 |  |  |  |  |  |  |
| 时间2 |  | 安慰剂组 |  |  |  |  |  |  |
|  |  | 中药香槟方组 |  |  |  |  |  |  |
| ： |  | ： |  |  |  |  |  |  |
| ： |  | ： |  |  |  |  |  |  |
|  |  | ： |  |  |  |  |  |  |
|  |  | ： |  |  |  |  |  |  |
| 最后 |  | 安慰剂组 |  |  |  |  |  |  |
|  |  | 中药香槟方组 |  |  |  |  |  |  |
| 总计 |  |  |  |  |  |  |  |  |

表32 两组术后出现各种并发症情况

| 观察时点 | 并发症 | 安慰剂组 | 中药香槟方组 | 总计  （%） |
| --- | --- | --- | --- | --- |
| 术后  第1天 | 肺感染 |  |  |  |
|  | 伤口感染 |  |  |  |
|  | 伤口裂开 |  |  |  |
|  | 尿路感染 |  |  |  |
|  | 下肢深静脉血栓 |  |  |  |
|  | 其他 |  |  |  |
| 术后  第2天 | 肺感染 |  |  |  |
|  | 伤口感染 |  |  |  |
|  | 伤口裂开 |  |  |  |
|  | 尿路感染 |  |  |  |
|  | 下肢深静脉血栓 |  |  |  |
|  | 其他 |  |  |  |
| **：** | **：** |  |  |  |
| **：** | **：** |  |  |  |
| **：** | **：** |  |  |  |
| **：** | **：** |  |  |  |
| **：** | **：** |  |  |  |
| **：** | **：** |  |  |  |
| 术后第7天 | 肺感染 |  |  |  |
|  | 伤口感染 |  |  |  |
|  | 伤口裂开 |  |  |  |
|  | 尿路感染 |  |  |  |
|  | 下肢深静脉血栓 |  |  |  |
|  | 其他 |  |  |  |
|  | 合计 |  |  |  |

**表33 分组死亡情况**

| 死亡 | 安慰剂组 | 中药香槟方组 | 总计  n（%） |
| --- | --- | --- | --- |
| 有 |  |  |  |
| 无 |  |  |  |

**患者记录卡**
